# Field-Portable Device for Detection of Controlled and Psychoactive Substances from e-Cigarettes

**DOI:** 10.1021/acsomega.4c08614

**Published:** 2025-02-17

**Authors:** Matthew Gardner, Celeste Bowden, Shoaib Manzoor, Gyles E. Cozier, Rachael C. Andrews, Sam Craft, Martine Skumlien, Peter Sunderland, Tom Tooth, Peter Collins, Alexander Power, Tom S. F. Haines, Tom P. Freeman, Jennifer Scott, Oliver B. Sutcliffe, Richard W. Bowman, Stephen M. Husbands, Christopher R. Pudney

**Affiliations:** † Department of Life Sciences, University of Bath, Bath BA2 7AY, U.K.; ‡ Department of Psychology, University of Bath, Bath BA2 7AY, U.K.; § Avon and Somerset Police, Valley Road, Bristol BS20 8JJ, U.K.; ∥ Department of Computer Science, University of Bath, Bath BA2 7AY, U.K.; ⊥ Centre for Academic Primary Care, Bristol Medical School, University of Bristol, Bristol BS8 2PS, U.K.; # MANchester DRug Analysis & Knowledge Exchange (MANDRAKE), Department of Natural Sciences, Manchester Metropolitan University, Manchester M1 5GD, U.K.; ¶ School of Physics and Astronomy, University of Glasgow, Glasgow G12 8QQ, U.K.; ∇ Centre for Bioengineering and Biomedical Technologies, University of Bath, Bath BA2 7AY, U.K.

## Abstract

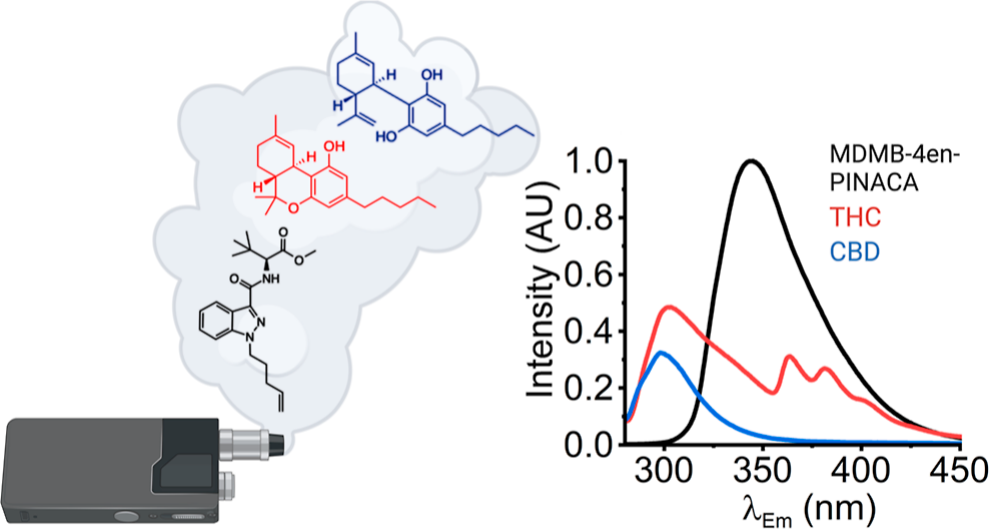

Synthetic cannabinoids (SCs) are novel psychoactive substances (NPS) that are highly potent and associated with a range of severe toxicities. SC use, which is common in UK prisons and homeless communities, typically involves combustion of SC-soaked herb or paper material. Recently, e-cigarettes (or vapes) have emerged as popular delivery vehicles for SCs, and consumption among the general population has risen significantly. SC-containing e-cigarettes (or e-liquids) are typically sold as imitation cannabis-containing products and carry increased risk of adverse outcomes including psychosis, seizure, and cardiac arrest. Numerous incidences of overdose have been reported in UK schools. SCs cannot be identified in complex e-liquid matrices using current field-portable detection technologies, preventing the rapid screening of suspicious products. Herein, we report on the design and development of a device that can rapidly detect SCs and other relevant drugs in sealed e-cigarettes and e-liquids. We describe and implement a method to artificially actuate an e-cigarette, simultaneously depositing e-liquid vapor onto a physical matrix. We couple this extraction method with fluorescence-based detection to create a rapid and generic test for SC-containing e-cigarettes. In addition, we expand the potential of the detection modality by leveraging the photochemical degradation of THC and nitazenes on a solid matrix as a means for their detection from e-liquids and sealed e-cigarettes. We show that SCs, delta-9-THC and nitazenes can be detected in e-liquid from concentrations 0.2, 5, and 1.5 mg/mL, respectively.

## Introduction

Synthetic cannabinoids (SCs) are a class of designer drugs that replicate the physiological effects of tetrahydrocannabinol (THC) via binding to cannabinoid receptors CB1 and CB2.^1^ Several structural classes of SCs have entered the illicit drug market, arising from iterative structural diversification around a common architecture.^2^ Physiological effects of newer SCs are divergent from early compounds synthesized as cannabimimetics, whereby complex interactions outside of CB1/CB2 agonism have been reported.^3^ Notably, these off-target effects may be responsible for the toxicity reported for a wide range of SCs.^4^

**Figure 1:**
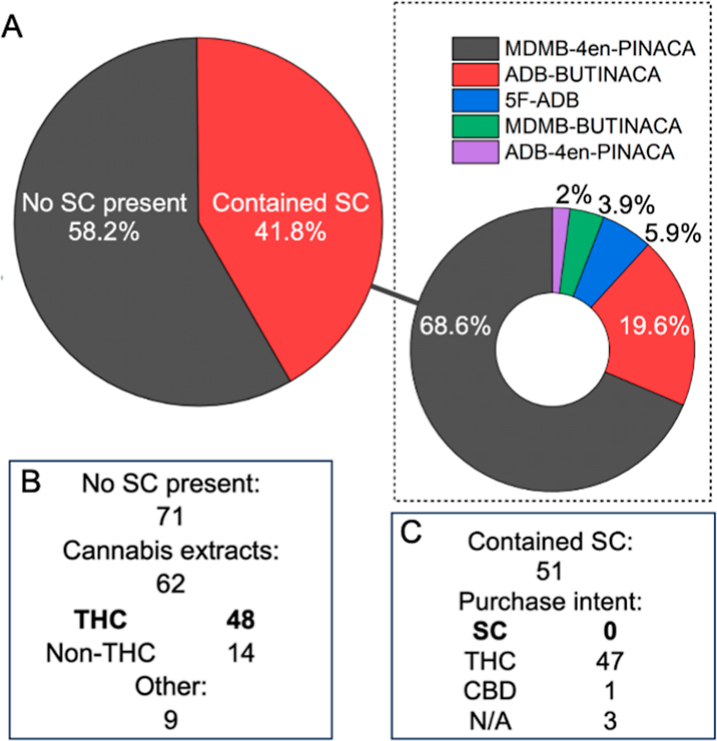
Prevalence of SC-containing e-cigarettes. (A) Analysis of 122 e-cigarettes and e-liquids submitted to UK drug checking service WEDINOS between January 2023 to April 2024. (B) Breakdown analysis of non-SC containing submissions according to drug content. (C) Breakdown analysis of SC-containing submissions according to purchase intent.

In the UK, SC consumption is prevalent in the homeless community, where SCs are smoked in herbal preparations, and in the prison estate, where SC-soaked paper is smuggled in as contraband.^5−7^ Recently, however, e-cigarettes have emerged as a popular delivery vehicle for SCs, consistent with their rise in popularity as nicotine delivery devices among young people.^8,9^ E-cigarettes appeal to young people through attractive packing and availability in a wide variety of flavors, while also providing an unsuspicious route of drug administration.^10,11^ Indeed, a major demographic for SC-containing e-cigarettes appears to be school age children. 31% of children up to age 17 have tried cannabis,^12^ meaning dealers can exploit the appeal of e-cigarettes to mis-sell SC-containing e-liquid as cannabis extract.^13^ Data from the UK drug checking service WEDINOS shows that 41% of 122 drug-laced e-cigarettes submitted for testing between January 2023 and April 2024 contained SCs.^14^ Notably, none of these samples were submitted with the purchase intent of SCs (Figure 1A–C). As SCs are more potent than THC and present in e-liquid at unknown quantities, the risk of overdose is higher, with potentially severe side-effects including respiratory depression and cardiac arrest.^15^ Further, recent work demonstrates that SC e-cigarettes are becoming less expensive, with a median cost of as £3.39/mL, and as little as £1.60/mL; cheaper per unit volume than a commercial e-cigarette.^16^

Nitazenes are a series of highly potent, fully synthetic opioids that have recently emerged into the illicit drug supply and has led to many deaths throughout the world.^17^ Nitazenes are usually added to counterfeit tablets (i.e., benzodiazepines) or combined with heroin, where numerous incidences of overdose have been reported due to unintentional consumption.^17−19^ Recent media reports are also emerging of e-cigarettes spiked with nitazenes, in addition to a WEDINOS submission from November 2022.^14,20,21^ An overdose death from opioid toxicity associated with the use of a nitazene-containing e-cigarette has also been reported in Australia.^22^ Given their high risk, we suggest the presence of nitazenes in e-cigarette liquid is worthy of detailed monitoring.

Established field-portable drug detection technologies such as Raman and FTIR are designed to work primarily on formulations where compounds of interest are present at a high purity.^23−25^ These instruments perform poorly on mixtures containing a low concentration of active compound, meaning detection of drugs including SCs directly from e-liquid is not feasible.^26^ Lab-based testing approaches (i.e., GC–MS) are used as the gold standard for assessing drug content of suspicious e-cigarettes, but are limited by long turnaround time, primarily restricting use to low-throughput evidentiary analysis.^27−29^ We were motivated by the current lack of point-of-care testing technology to develop a device that can rapidly report on drug content of sealed e-cigarettes and e-liquids liquids, to be deployed to inform urgent care.

We have previously implemented generic detection of SCs on a range of physical matrices in an ultraportable, hand-held device.^30^ Here, we report the development of a device that can extract e-liquid from suspicious e-cigarettes through artificial actuation and simultaneous deposition onto a solid matrix. We couple this novel extraction method with our previously reported generic detection to inform on SC content of sealed e-cigarettes rapidly and nondestructively. Building on this, we also detail a novel method for detection and discrimination of THC and nitazenes in sealed e-cigarettes using photochemical degradation on a solid matrix. We anticipate this field-portable device will be deployed as an effective harm reduction measure in the hands of trained school-staff, community support workers and as part of community drug checking services.

**Figure 2:**
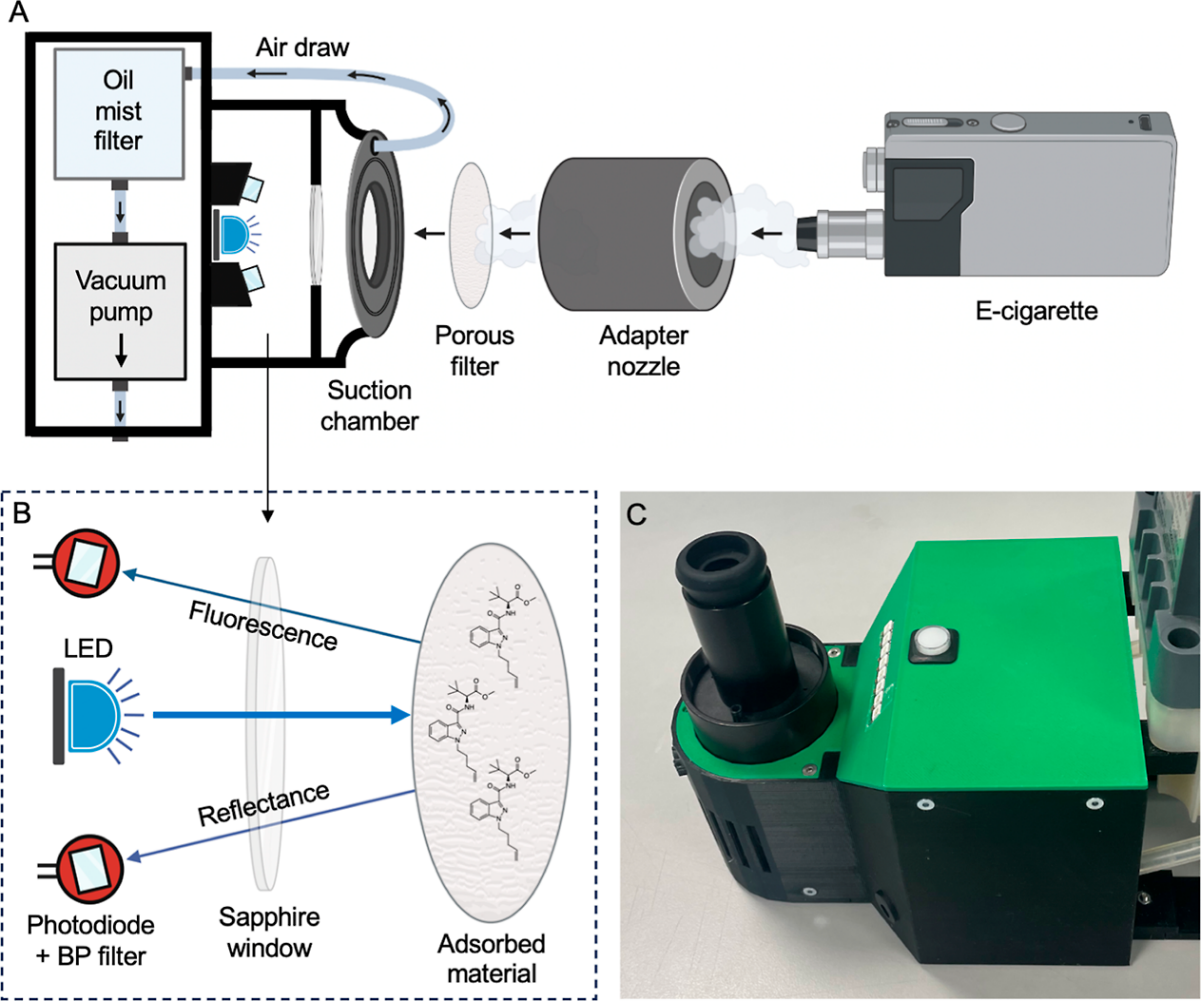
E-cigarette drug detection device (EDD). (A) Device schematic showing key functional components and direction of air draw from vacuum pump to suction chamber via flexible plastic tubing. (B) Enlarged view of SC detection apparatus comprising two amplified photodiodes (PD) with bandpass filters and 265 nm LED for filter irradiation. PD C310 is centered at 310 nm with a 10 nm bandwidth and C350 is centered at 350 nm with a 50 nm bandwidth. Detection apparatus is essentially as we describe in ref (30). (C) Functional prototype device housed in 3D-printed shell. Figure created with BioRender.

Previous work has shown SCs exhibit relatively strong fluorescence emission, with a prominent emission band centered at ∼350 nm that is conserved across all major structural classes.^30^ When adsorbed onto physical matrices (i.e., paper), SC emission can be deconvolved from background autofluorescence with irradiation from a sufficiently bright UV–C light source.^30^ In E-cigarettes, SCs are present in a heterogeneous e-liquid vehicle that consists of propylene glycol (PG), vegetable glycerin (VG), flavoring compounds and nicotine.^31^ This e-liquid is not directly removable from many types of e-cigarette pods for subsequent analyses. E-cigarette actuation is draw activated; inhalation is detected by an airflow sensor that responds by heating an atomizer coil to vaporize surrounding e-liquid.^32^ To extract e-liquid nondestructively, we have developed a device that can artificially actuate an e-cigarette while simultaneously depositing its vapor onto a physical matrix. We have paired this extraction approach with in-line optical elements capable of performing generic detection of SCs (as in ref (30)), to rapidly inform on drug content. A schematic of the device is shown in Figure 2A.

## Results and Discussion

The device consists of a universal e-cigarette adapter, a suction chamber, pumping/filtration components and optical detection elements. An e-cigarette is inserted into the universal adapter that when interfaced with the suction chamber forms an airtight assembly. Activation of the vacuum pump via a button press draws air through these components and the inserted e-cigarette, causing actuation. Vapor is deposited on a porous filter sandwiched in a holder between the suction chamber and the e-cigarette adapter. Deposited e-liquid material is irradiated on-filter by a high-powered (80 mW) 265 nm LED, temperature regulated by a fan and heat sink (Figure 2B). Reflectance and fluorescence components arising from filter irradiation are captured by photodiodes, with fitted bandpass (BP) filters for wavelength selection, similar to our previous report.^30^ The photodiodes are configured so that the optical path and focal lengths align with the filter holder for optimal light collection. LED irradiation is performed though a sapphire window embedded into the base of the suction chamber. The device is housed in a 3D-printed shell as shown in Figure 2C. The detachable e-cigarette adapter located at the front of the device is interfaced with the suction chamber and filter holder through a quick-lock mechanism. The device is engaged with a single button press and an LED strip indicates stages of function and eventual outcome of e-cigarette testing. The addition of an oil mist filter (Figure 2) mitigates the risk of exposure to controlled substances when using the device.

**Figure 3:**
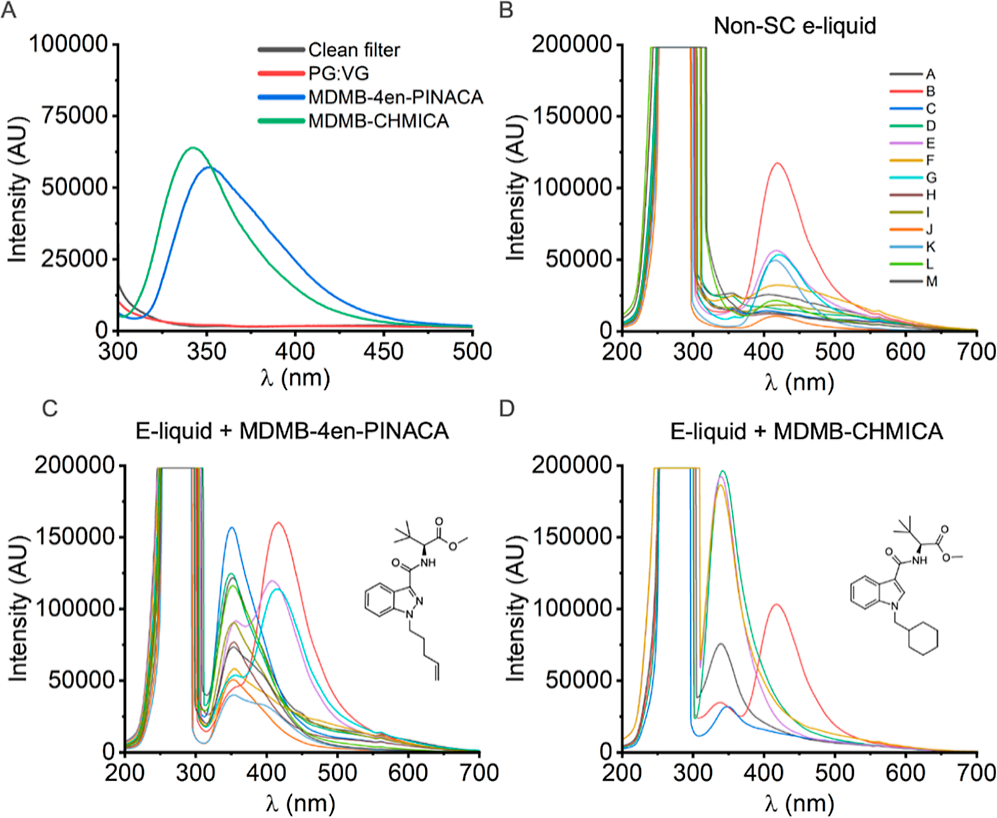
On-filter spectroscopic evaluation of e-liquid vapor. (A) Spectra of clean Whatman 42 (2.5 μM pore size) filter paper and filters with deposited vapor from artificially actuated e-cigarettes containing: PG/VG vehicle alone (50:50 v/v), 1.5 mg/mL MDMB-4en-PINACA in PG/VG, 1.5 mg/mL MDMB-CHMICA in PG/VG. Contributions from LED reflectance removed. Integration times 500 ms (B), spectra of 13 exemplar commercially available e-liquids artificially actuated and deposited onto filters. E-liquids used are described in Table S1. (C) Corresponding e-liquids containing 1.5 mg/mL MDMB-4en-PINACA. Spectra recorded with integration times set between 4000 and 500 ms and matched across unaltered and SC-containing e-liquid. (D) Deposited e-liquids containing 1.5 mg/mL MDMB-CHMICA. Integration times 4000 ms. Corresponding unaltered e-liquids are shown in Figure S2A. E-liquids used are described in Table S2.

To ensure airflow drawn by the vacuum pump would be sufficient for e-cigarette actuation, we selected porous filter paper as a matrix for vapor deposition. We reasoned that a pore size roughly equivalent to the diameter of e-liquid aerosols (0.3–3 μM) would maximize the quantity of potential drug material adsorbed during actuation.^33^Figure S1A,B shows that a 2.5 μM pore size filter performed best for both indole- and indazole-based SCs, thus was selected for use. Ashless paper was selected for use in the device as negligible autofluorescence was detected upon irradiation with a 265 nm LED (Figure 3A). E-cigarettes are designed with an integrated timer that cuts battery power at ∼10 s to prevent coil damage. We have designed our device to perform a 20 s pumping step, incorporating ∼10 s actuation and filter “drying” stages. We find the additional drying stage has a homogenizing effect on adsorbed material as it is pulled though the porous filter, providing consistent optical detection. Mass of aerosol deposited onto filters from a range of single use e-cigarettes was measured as 18.8 ± 3.3 mg (*n* = 12). We note that successful detection is dependent upon total mass of material deposited on the filter, not total puff duration.

Quantitative analysis of SC-containing e-liquids in the literature suggests a concentration range of ∼1–25 mg/mL, compound dependent.^34^ To assess our ability to detect at real-world concentrations, SC-containing e-cigarettes were prepared and artificially smoked using the e-cigarette drug detection device (EDD). Direct spectral measurements were performed on removed filters using a UV–vis spectrometer and a 265 nm LED, identical to the EDD light source. Measurements were taken on the side of the filter facing optical detection elements, rather than the e-cigarette mouthpiece. Figure 3A shows the spectra acquired after artificial actuation of, PG/VG-only, MDMB-4en-PINACA and MDBM-CHMICA containing e-cigarettes. Fluorescence emission of both indazole- and indole-based SCs is resolved on-filter when present in PG/VG e-liquid at 1.5 mg/mL, with a λ_Em_^max^ of ∼350 nm. No fluorescence emission is detectable from actuation of an e-cigarette containing PG/VG only. These data indicate that we can detect SCs present in e-liquid at concentrations represented in the low range of real-world samples.

In the EDD, measurements are recorded on known matrix with negligible autofluorescence. Resolving SC fluorescence from that of codeposited flavoring compounds presents the greatest challenge to detection. Direct spectral measurements were performed on filters removed after actuation of e-cigarettes containing a range of commercially available e-liquid, as to assess the impact on SC emission. From Figure 3B, of 13 exemplar e-liquids, the major emission band from flavoring compounds is localized in the spectral region ∼400–450 nm, red-shifted from SC emission by ∼50 nm. On comparing to spectra acquired for identical e-liquids containing 1.5 mg/mL MDMB-4en-PINACA, we see that SC emission is resolved from that of the flavoring compounds (Figure 3C). We note that in some instances, emission is convolved of SC and flavoring compounds; however, a spectral feature is still present at ∼350 nm. Figure 3D shows how indole-based SC fluorescence is also resolved in the presence of flavoring compounds, with spectra of corresponding unaltered e-liquids shown in Figure S2A. We posit that the variation in magnitude of SC emission is due to differential quenching occurring in e-liquid variants with different chemical composition. Taken together, these data indicate that sensitive, on-filter detection of SCs in the heterogeneous mixture of commercial e-liquid is possible with the EDD.

**Figure 4:**
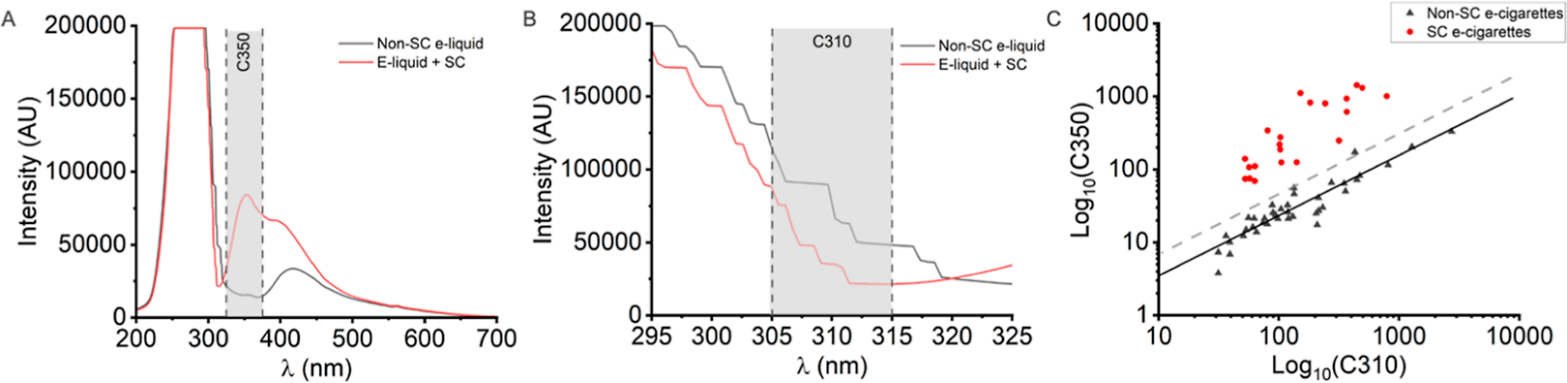
Predictive model for detection of SC in e-liquids. (A) Averaged spectra of 13 exemplar unaltered and SC-containing e-liquids shown in Figure 3B,C. Spectral region and bandwidth of PD C350 highlighted with gray shading. (B) A with C310 highlighted in gray shading. (C) Relationship of C310 and C350 values, plotted on a Log_10_ scale. PD values recorded from direct device measurement of 60 e-cigarettes, 21 containing 1.5 mg/mL SC and 39 unaltered. The black line is a power function fitted through the unaltered e-liquid data and the gray dotted line shows the scaling of this function to give a false positive rate of ∼5%.

We next assessed the response of bandpass filters C310 (310 ± 5 nm) and C350 (350 ± 25 nm) to establish a numerical detection model for SCs present in e-liquid, similar to the principle of our previous detection method.^30^ Our previous work^30^ shows that the ratio of these spectral regions can be modeled to establish numerical model for alarm thresholding. A total of 60 e-cigarettes were artificially actuated and measured with the EDD, 39 containing unaltered e-liquid and 21 containing 1.5 mg/mL SC (MDMB-4en-PINACA × 15, MDMB-CHMICA × 6) e-liquid. Figure S2D shows the averaged response of PDs C310 and C350 for both SC and unaltered e-liquids. When SCs are present the average response of C350 rises from 46 to 481 arbitrary units, this increase is visualized spectroscopically in Figure 4A and S2B, where contributions from SC emission alone are responsible for increased signal magnitude in the spectral region of C350 (325–375 nm). Inversely, presence of SCs in e-liquid reduces the average magnitude of the C310 response, dropping from 261 to 206 when SCs are present. This change is visualized in Figures 4B and S2C and arises through a decrease in reflected light from loaded filters. We suggest this is predominantly caused by the relative increase in absorption from ∼280 to 320 nm when SC are present in e-liquid (Figure S3).

Figure 4C shows a log-scaled plot of C310 and C350 values obtained for each of the 60 e-liquid samples. Fitting of a power function (black line) through the unaltered e-liquid data provides a means to calculate predicted C350 background values (C350Pred) for a given C310 reading. Upon measurement of deposited e-liquid on-filter, C350 values recorded over C350Pred will cause the device to indicate for presence of SCs. We have empirically adjusted scaling of the power function through unaltered e-liquid data to give a ∼5% false positive rate, as shown by the gray dotted line. We note the superiority of incorporating both C310 and C350 PD responses into a numerical model over relying on absolute magnitude chance in C350 to detect presence of SCs. As mentioned above, SC emission is differentially quenched in a range of e-liquids, however this model allows for dynamic background prediction of e-liquid measured, to provide a sensitive means of SC detection. Figure S4 shows the validation of our predictive model as a means for detecting SCs in e-liquid, using real world, police seized samples.

### Photochemical Sensing of THC and Nitazenes

**Figure 5:**
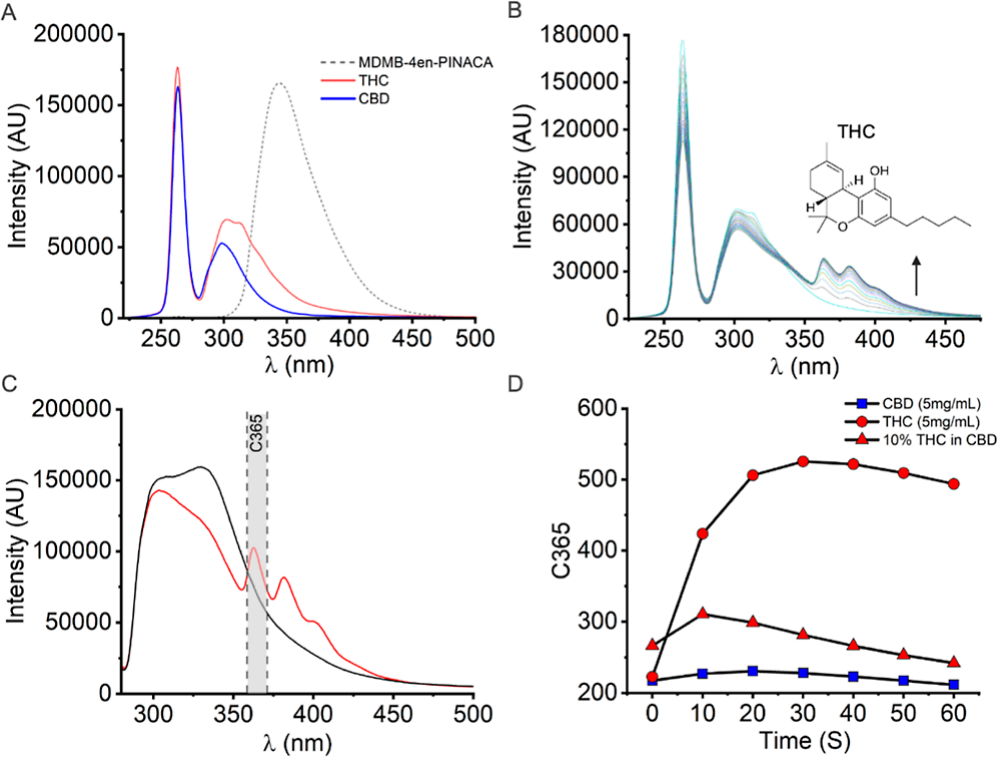
Photochemical degradation of THC as a basis for detection in e-liquid/e-cigarettes. (A) Spectra of MDMB-4en-PINACA (1 mg/mL), THC (0.1 mg/mL) and CBD (0.1 mg/mL) in 1 mL ethanol. Irradiation at 265 nm. Integration times 500 ms, 3000 and 1500 ms, respectively. (B) Time–course measurement of THC [as in (A)] emission. Spectra were recorded from 0 to 180 s in 10 s increments. (C) Spectra obtained from 0.5 mg THC (100 μL of 5 mg/mL in PG/VG) deposited on Whatman 42 filter. Black line indicates measurement at *t* = 0 s, red line indicates measurement at *t* = 30 s. Integration time 4000 ms. The spectral region and bandwidth of Photodiode C365 is highlighted in gray. (D) Time–course response of C365 obtained for 5 mg/mL CBD, 5 mg/mL THC and 10% THC in CBD (5 mg/mL THC, 50 mg/mL CBD) e-cigarettes with constant 265 nm irradiation.

From Figure 1B, the majority of non-SC containing e-cigarettes/e-liquids submitted for drug checking contained THC. Also, 92% of SC-containing e-cigarettes were purchased with the intent of receiving THC. E-cigarettes as a route of administration for cannabis are most popular among young people, where modified commercial devices are often used.^35^ Rapid detection of THC from sealed e-cigarettes and e-liquid is thus desirable, where discrimination from SCs provides important harm identification. Therefore, we assessed the potential for specific identification of THC using the EDD. Figure 5A shows fluorescence spectra of THC, CBD and MDMB-4en-PINACA collected in ethanol and irradiated with 265 nm LED. The emission band of CBD and THC are centered at ∼300 and 310 nm, respectively. In addition, the emission spectra are structurally different, at least at the same concentration, with THC having a bidistributed spectral maximum.

The spectral features of both CBD and THC align approximately to the spectral region captured by C310 (particularly for THC). However, irradiation of these species deposited on-filter would cause the emission band to become convolved with LED reflectance, due to the high-power of the light source and sensitivity of PDs. Moreover, distinguishing the spectral features of CBD and THC would be potentially challenging given their significant overlap. We therefore considered an alternative approach.

We have previously described photochemical fingerprinting as an effective tool for structural discrimination of individual SCs.^37^ That is, inducing a photochemical change in the analyte and using the altered emission profile as a characteristic presumptive identifier of the analyte. Speculatively, we were interested to explore if a similar phenomenon could support differential detection of CBD and THC. Figure 5B shows a time–course plot of spectra collected for THC in EtOH at 0.1 mg/mL. We find that continuous irradiation at 265 nm causes the emission band of THC to rapidly undergo a notable structural change, where three new spectral features emerge centered at ∼365, ∼380 and ∼405 nm. It is logical to suggest that the observed shift in emission arises from a photochemical reaction that results in production of new fluorescent species. Moreover, from Figure S8, CBD does not produce similar, or indeed any new spectral features, at least under the conditions used here. That is, under the same conditions, the emission band of CBD remains unchanged, thus providing a means to discriminate the two cannabinoids. This is serendipitous because, at least in the UK, CBD is legal and THC is illegal.

We next investigated whether the photochemical reactivity of THC is preserved when adsorbed onto a physical matrix. Figure 5C shows spectra recorded from 0.5 mg THC deposited onto a Whatman 42 (2.5 μm pore size) filter, as used in the EDD. This quantity represents the low-end of what might be expected from a single actuation of real-world THC e-cigarettes, where concentrations of 500 mg/mL and above are typical. From Figure 5C, the structure and center of mass of the THC emission spectrum in EtOH is broadly preserved. Irradiation of the sample similarly gives rise to spectral features consistent with solution measurement, suggesting the same photochemical process is active and traceable when deposited on a solid matrix.

To exploit the photochemical reactivity of THC as a means of detection, we added an additional PD that would capture changes in emission structure postdegradation (C365 region shown in Figure 5C; 365 ± 5 nm). The new spectral feature centered at ∼365 nm has both the largest relative magnitude and is the most blue-shifted species from the emission spectra arising from flavoring compounds (Figure 3). This means that the C365 PD minimizes any artifactual spectral convolution.

**Figure 6:**
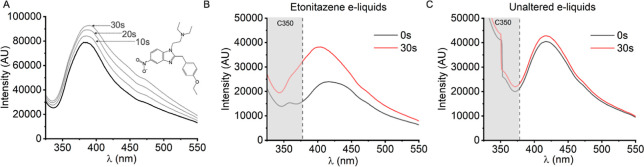
Nitazene detection in e-liquid is possible through photochemical degradation. (A) Spectra of deposited vapor on Whatman 42 filter from artificially actuated e-cigarette containing 1.5 mg/mL etonitazene (freebase) in PG/VG. Integration time 4000 ms. LED peak chopped. (B) Averaged spectra acquired from e-cigarettes containing 1.5 mg/mL etonitazene (freebase) in a selection of 9 commercially available e-liquid flavors. Integration time 4000 ms. The specific e-liquids used are described in Table S3. (C) As in (B), with identical selection of unaltered e-liquids.

Figure 5D shows the time–course plot of the C365 response, recorded from testing of a series of cannabinoid-containing e-cigarettes prepared in house. As expected, a clear increase in C365 magnitude for an e-cigarette containing 5 mg/mL THC is observed, with no similar change for an e-cigarette containing an equivalent concentration of CBD. It is worth noting that this is an extremely low concentration compared to the practical use of THC. Figure 5D also shows that detection of low concentrations of THC (10%) in the presence of CBD is also possible with this approach. From Figure 5D, we find that the most significant changes to C365 signal are apparent at between ∼10 and 30 s, with subsequent decay of the signal. We suggest the decay of the signal represents a subsequent photochemical degradation step to a nonemissive photoproduct. Clearly this kind of time frame suits the type of rapid detection we envisage. Thus, to detect THC with the EDD, we employ a 10 s irritative step where the calculated ratio of C365 response pre- and postirradiation must exceed a predefined threshold. Cannabinoid vape cartridges contain a concentrated resin of active compounds extracted from plant material using organic solvents. To assess whether the presence of these components affect detection of THC, we tested the device on real-world cannabinoid extract products. Figure S9 shows the validation of photochemical-based THC detection from a range of vape pens seized by the police. Furthermore, semisynthetic cannabinoids (SSCs), chemically modified cannabinoid derivatives, are increasingly being identified in e-cigarettes. Hexahydrocannabinol (HHC) and delta-8-THC are two major SSCs with psychotropic effects that mimic delta-9-THC.^36^ We note that detection of delta-8-THC will be possible with the photochemical approach described above but not HHC, which like CBD, is not photochemically reactive (Figure S10). As above, nitazenes are highly potent synthetic opioids.^17^ While nitazenes, are not at present found regularly in e-cigarette liquid, their high risk means detection would be useful. To pre-empt the detection of this very high-risk scenario, we investigated a means for their detection in e-liquid using the EDD. The recency of nitazene presence in the dug supply means the literature regarding nitazene concentration in e-liquid, or indeed oral potency, is absent, and so we have investigated similar concentrations to our SC experiments above. Figure 6A shows the spectrum acquired directly on-filter after artificial actuation of a 1.5 mg/mL etonitazene e-cigarette. The solid black plot shows emission captured at *t* = 0 s, with the three gray plots showing emission after constant irradiation with the 265 nm LED for 10, 20 and 30 s. The main emission band of etonitazene is centered at ∼390 nm. This presents a challenge to detection in many types of e-liquids as etonitazene emission overlaps significantly with the emission bands of flavoring compounds. However, similar to THC above, etonitazene apparently undergoes a photochemical reaction that results in an increased magnitude of emission and a slight red-shit to ∼400 nm, most apparent at *t* = 30 s under the conditions used here.

To investigate if it was possible to leverage this apparent photochemical response for detection, we assessed the time-course change of etonitazene emission in the presence of a series of commercially available e-liquids. Figure 6B shows the averaged spectrum of 9 e-liquids containing 1.5 mg/mL etonitazene, deposited onto filters through artificial e-cigarette actuation. We see a significant increase in magnitude at *t* = 30 s in the spectral region 350–450 nm, indicative of etonitazene photodegradation. No such change is observed in unaltered vape liquid (Figure 6C) in this spectral window, indicating flavoring compounds are not photochemically active under the conditions used here. Therefore, although etonitazene fluorescence may be convolved with those of flavoring compounds, photochemical degradation provides a means for detection in a similar manner to the detection of THC above. PDs C350 and C365 can be used to monitor changes in nitazene emission magnitude as their spectral windows align to the blue end of the pre- and postirradiation feature. Incorporation of nitazene detection into the EDD is thus accomplished by setting a threshold for change in magnitude of both C350 and C365 response from *t* = 0 to *t* = 30 s. Moreover, Figure S11F shows a time–course plot for metonitazene, showing a similar change in emission on irradiation. Given, the core aromatic moiety of nitazenes are the same (a benzimidazole), and our evidence implies the approach works similarly on different nitazenes, we suggest this method could be generically applied to nitazene discrimination in e-cigarettes.

## Conclusions

Herein, we have built on our previous SC detection methodology to enable detection in e-cigarette liquid and directly from sealed e-cigarettes. This advance is timely, given the apparent prevalence of SC e-cigarettes being sold as “THC”. That is, this implementation of our technology has an immediate application in both tracking the occurrence of SC e-cigarettes and providing harm reduction information. We have demonstrated that this technology is amply capable of detecting the low published range of SC concentrations found in e-cigarettes. Given we did not find a single false negative from our sample set in Figure 3, there is ample potential for tuning the detection limit, with a trade-off between sensitivity and specificity as with all technologies of this type.

Clearly, the context of the detection of SC e-cigarettes is not complete without the capability to detect THC. To that end, we have leveraged the novel finding that THC can be driven to generate a fluorescent photoproduct that is distinct from CBD, and serendipitously, this detection methodology can be incorporated into our EDD technology, with minimal adjustment.

We reasoned that other drugs might similarly be detectable from a fluorescence signal. Some of the most significant concern at present (at least in the UK) comes from the increasing reports of overdoses with synthetic opioids (nitazenes). Sadly, these drugs appear to be responsible for a large number of deaths in the heroin using community in the UK and the concern is that they become a feature of the wider drug supply. We have therefore explored the potential to detect nitazenes in e-cigarette liquid. We find similar to THC, that combining fluorescence detection with photodegradation yields a novel signal that can be identified even in e-cigarette liquid. While we acknowledge nitazene containing e-cigarettes are sparsely reported the UK at this time, and so we do not have real world seizure data, these findings support the notion that the detection is feasible.

## Materials and Methods

### e-Cigarette Drug Detection Device

Device design is described in the main text (Figure 2). The e-cigarette adapter and vacuum chamber assembly are constructed from threaded lens tubes (Thor Laboratories). Detection apparatus including: 265 nm LED (Thor Laboratories), photodiodes (Scitech instruments Ltd.) and sapphire window (Thor Laboratories) are off-the-shelf components. The device is controlled by Arduino nano RP2040 running a custom Circuit Python script. The Arduino, LED, photodiodes, vacuum pump and the LED indicator strip (Adafruit) are driven by a custom-made printed circuit board. The custom-made housing was 3D-printed in ABS plastic using an Ultimaker S3. The device uses Whatman 42, 2.5 μM pore size ashless filters for e-liquid drug detection.

### e-Cigarette Sample Preparation

In experiments involving artificial actuation of e-cigarettes, ELF BAR devices were filled with a diverse range of commercially available e-liquids. ELF BARs were disassembled to expose adsorbent sponge and wicking material components. These were cleaned with methanol and dried before adding fresh e-liquid, 2 mL for all experiments/e-liquid flavors. For SC-containing e-cigarettes, e-liquid was prepared at 1.5 mg/mL with either MDBM-4en-PINACA or MDMB-BINACA, where 300 μL 10 mg/mL SC in ethanol was mixed with 1700 μL e-liquid. In addition to experiments performed using modified elf bars, a rage of commercially available sealed vapes were used to construct our predicative model for SC-detection in deposited e-liquid.

### Fluorescence and Absorption Spectroscopy

Fluorescence spectra were captured using a microspectrometer (ST-VIS, Ocean Insight) housed in a custom-built 3D-printed ring with heat skink and a high powered 265 nm LED (Boston Electronics). The UV–C LED was driven at 700 mA using a Mightex LED driver. Fluorescence emission was focused directly into the entrance slit of the spectrometer by a collimating lens placed in the fiber-optic input connector. Liquid samples were analyzed in a 1 mL crucible made from 3D printed black plastic (nonfluorescent and chemical resistant). Spectral acquisition on Whatman filters containing deposited e-liquid was performed by placing the spectrometer and LED assembly directly on top. Filters were analyzed with on the side facing away from the e-cigarette mouthpiece, as to validate the in-line detection approach employed by the device. Spectra were not averaged, or background subtracted.

Absorption measurements were performed using a UV–visible spectrophotometer (Agilent Technologies Cary 60). Temperature was maintained at 20 °C with Peltier accessory. Quartz fluorescence cuvettes were used to collect absorbance spectra for solutions of 1 mL sample volume. E-liquid samples and pure PG/VG containing SCs were diluted in EtOH and were background subtracted. Absorbance recorded between 800 and 200 nm with a scan rate of 600 nm/min and 1 nm intervals between data points.

### Extraction and Preparation of Drug Material

MDMB-4en-PINACA was extracted into ethanol from UK prison-seized crystal material of ∼81% purity. MDMB-CHMICA was extracted into ethanol from pure crystal. Etonitazene and metonitazene were prepared in house and confirmed as >95% pure (below). THC and CBD were purchased as standards. Highly pure (>80%) THC vape pens used in device validation were obtained from police seizures. Seized refill bottles of SC-containing e-liquid were obtained from police seizures.

### Compound Synthesis

Etonitazene and metonitazene were prepared using a previously published method.^38^ The ^1^H NMR, consistent with the etonitazene and metonitazene structure, are reproduced in Figures S12 and S13.

### NMR

The quantitative nuclear magnetic resonance (NMR) method used was based on a study that quantified SCs in seized e-liquids.^39^ Samples were prepared by mixing 200 μL of e-cigarette liquid with 400 μL of methanol-*d*_4_ (MeOD) containing 3 mg of 3-(trimethylsilyl)propionic-2,2,3,3-d_4_ acid sodium salt (purity ≥ 99%, isotopic purity 98 atom % D) (TSP). A set of MDMB-4en-PINACA concentrations (10–0.1 mg/mL) were prepared in 50:50 polyethylene glycol/glycerol (PG/VG) as standard e-cigarette liquids to test the quantification method.

^1^H NMR data were recorded on a Bruker AvanceCore 400 MHz spectrometer (1H frequency of 400.130), with a zg pulse sequence composed of 3.18 s acquisition time, 128 scans and 20 s delay. Chemical shifts were referenced to 3.31 ppm for residual CD_2_HOD solvent peak (from MeOD) and are reported in ppm. NMR spectra were processed with Mestralab Mnova 14.1 using automatic phase and Whittaker smoother baseline corrections, followed by zero filling (4× original size) and line broadening (1 Hz) to improve signal/noise ratio. Due to the large amounts of PG/VG in e-cigarette liquid, only the 4 peaks from the aromatic indazole core of the SCs could be reliably integrated. As many of these were used for the qNMR calculation, when not obscured by other additives in the e-cigarette liquid.

The following equation was used for the ^1^H qNMR quantitation
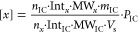
where [ ] is the concentration in mg/mL, *P* is the purity, n is the number of protons, Int is the integral value, MW is the molecular weight, *m* is the mass in mg, *V* is the volume in mL, IC is the internal calibrant, *x* is the analyte, and s is the sample. As the indazole peaks of the different SC compounds overlaid, when multiple SC compounds were present in each sample, the molecular weight of the main SC, as judged by the LC–MS chromatogram, was used in the calculation.

## Supporting Information Available

Impact of filter pore size on signal magnitude, spectra of example e-liquids and with SC present, photochemical sensitivity of CBD, THC and nitazenes, representative NMR spectra (PDF)

## Abbreviations

SC: synthetic cannabinoids
SO: synthetic opioid
