# Supplementary material for: Field-Portable Device for Detection of Controlled and Psychoactive Substances from e-Cigarettes
Source: ACS Omega. 2025 Feb 17;10(8):7839–47. doi: 10.1021/acsomega.4c08614 (PMC11886666; doi:10.1021/acsomega.4c08614)
Supplement: Supplementary file 1 — ao4c08614_si_001.pdf [file ao4c08614_si_001.pdf]

Supporting information. Automated detection of controlled substances from sealed e-cigarettes.

Matthew Gardner,[1,8] Celeste Bowden,[1] Shoaib Manzoor,[1] Gyles E. Cozier,[1] Rachael C. Andrews,[1] Sam Craft,[2] Martine Skumlien[2], Peter Sunderland,[1] Tom Tooth,[3] Peter Collins,[3] Alexander Power,[4] Tom S. F. Haines,[4] Tom P. Freeman,[2] Jennifer Scott,[5] Oliver B. Sutcliffe,[6] Richard W. Bowman,[7]* Stephen M. Husbands,[1]* Christopher R. Pudney[1,8]*

[1]Department of Life Sciences, University of Bath, BA2 7AY, UK, [2]Department of Psychology, University of Bath, BA2 7AY, UK, [3]Avon and Somerset Police, Valley Road, Bristol, BS20 8JJ, UK, [4]Department of Computer Science, University of Bath, BA2 7AY, UK, [5]Centre for Academic Primary care, Bristol Medical School, University of Bristol, Bristol, BS8 2PS, UK, [6]MANchester DRug Analysis & Knowledge Exchange (MANDRAKE), Department of Natural Sciences, Manchester Metropolitan University, Manchester, M1 5GD, [7]School of Physics and Astronomy, University of Glasgow, Glasgow, G12 8QQ, UK, [8]Centre for Bioengineering and Biomedical Technologies, University of Bath, Bath BA2 7AY, UK.

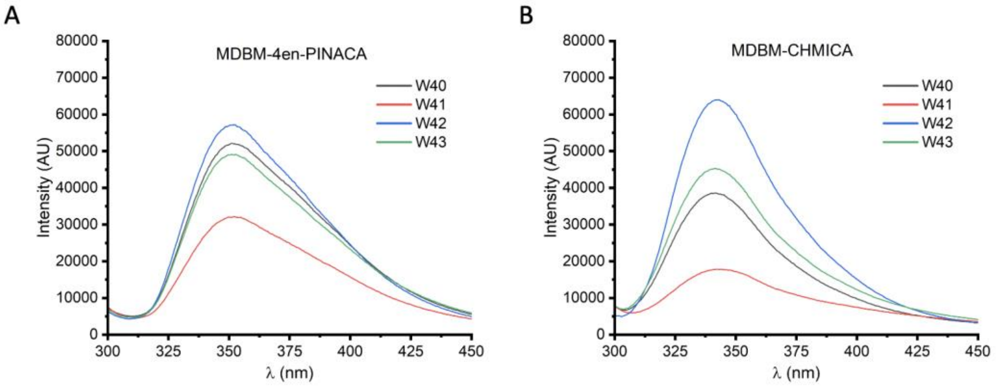

Figure S1. Impact of filter paper pore size on SC fluorescence signal. (A), Direct spectral measurement of Whatman filters 40-43 with deposited vapor from artificially actuated vapes containing 1.5 mg/mL MDMB-4en-PINACA in PG:VG. (B), As in A but with 1.5 mg/mL MDMB-CHMICA.

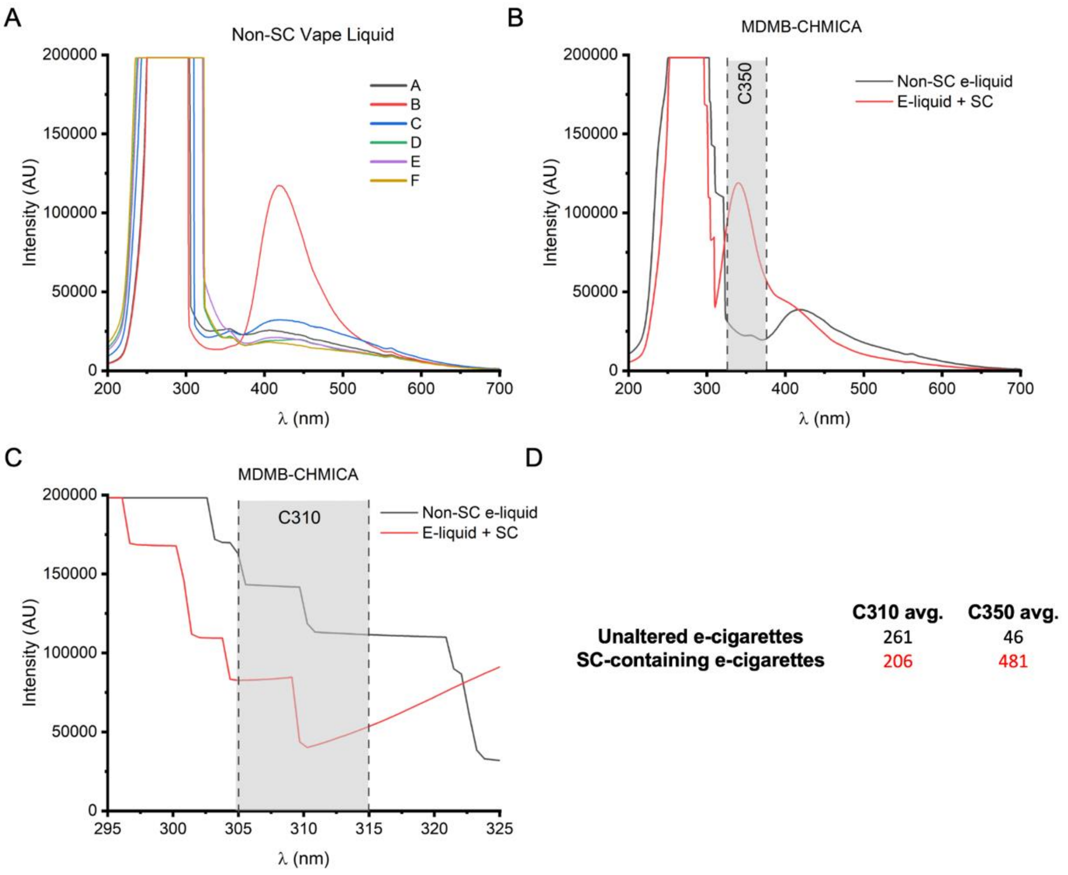

Figure S2. (A), Spectra of 6 exemplar commercially available e-liquids, artificially actuated and deposited onto filters. (B), Averaged spectra of 6 exemplar unaltered and SC-containing e-liquids shown in A and figure 3D. Spectral region and bandwidth picked up by PD C350 is highlighted with grey shading. (C), as in B, showing C310. (D), averaged response of PDs C310 and C350 for 21 SC-containing and 39 unaltered e-liquids.

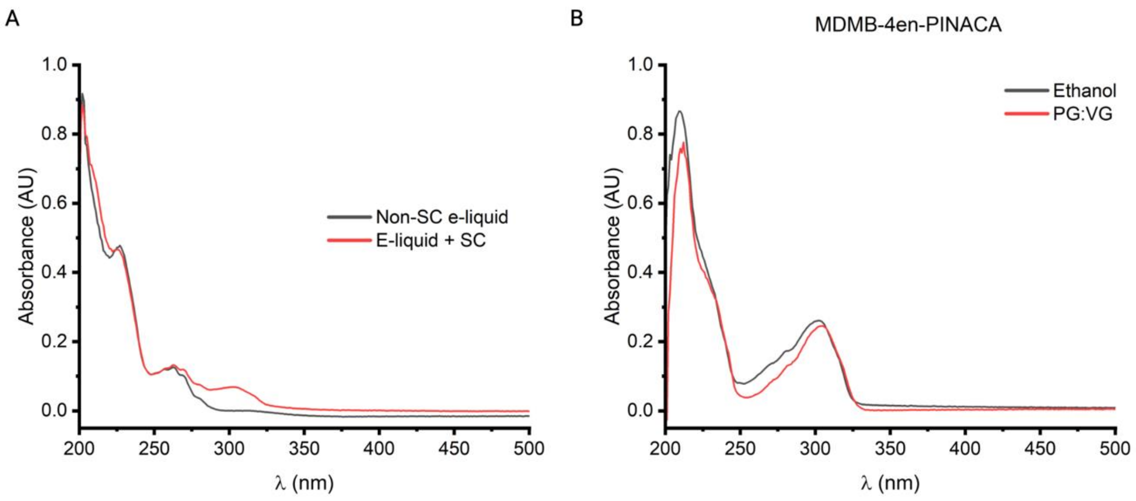

Figure S3. (A), Absorbance spectra of unaltered (non-SC) and 5 mg/mL MDMB-4en-PINACA e-liquid (R&M Tornado, Blue Razz). Both diluted 5000-fold in EtOH (Final concentration 1 μg/mL). (B), Absorbance spectra of MDMB-4en-PINACA at 5 μg/mL in both EtOH and PG:VG (50:50 v/v).

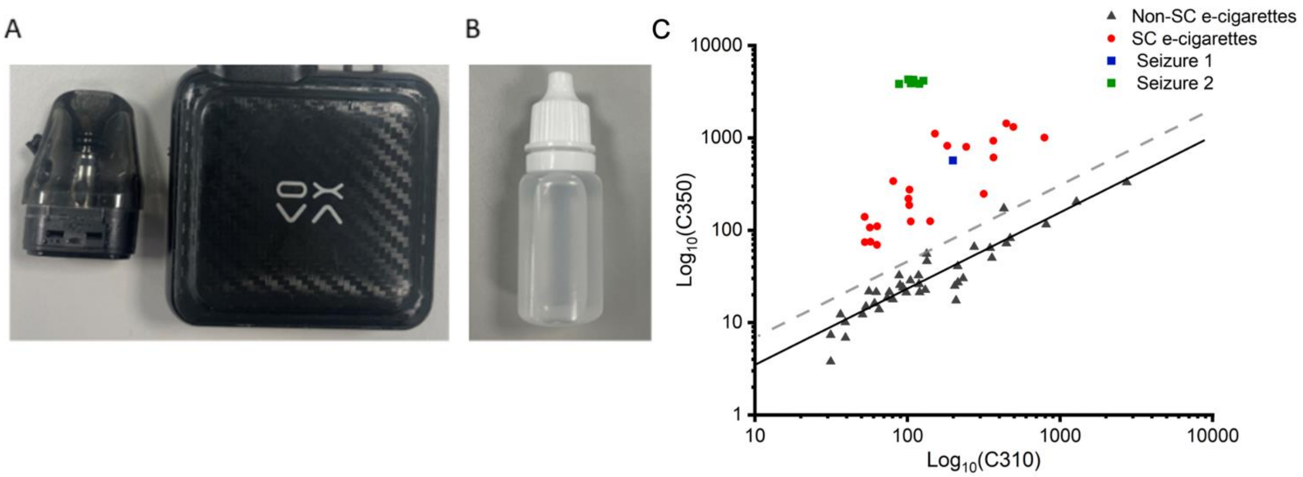

Figure S4. Validation of the predictive model for SC detection in e-liquid. (A), Refillable e-cigarette from Devon and Cornwall police, seizure 1. Extracted e-liquid was shown to contain 0.19 mg/mL MDMB-4en-PINACA by q1H NMR (Figure S6). (B), Exemplar e-liquid refill bottle from seizure 2. Mean concentration of seven identical bottles calculated as 0.90 mg/mL (SD = 0.05) by q1H NMR (Figure S7). (C), Plot of C310 and C350 values for eight samples (seizures 1 and 2) against numerical model for SC detection. The device indicated a positive result for all samples. Device measurements were performed by sampling 20μL of e-liquid onto Whatman filter discs.

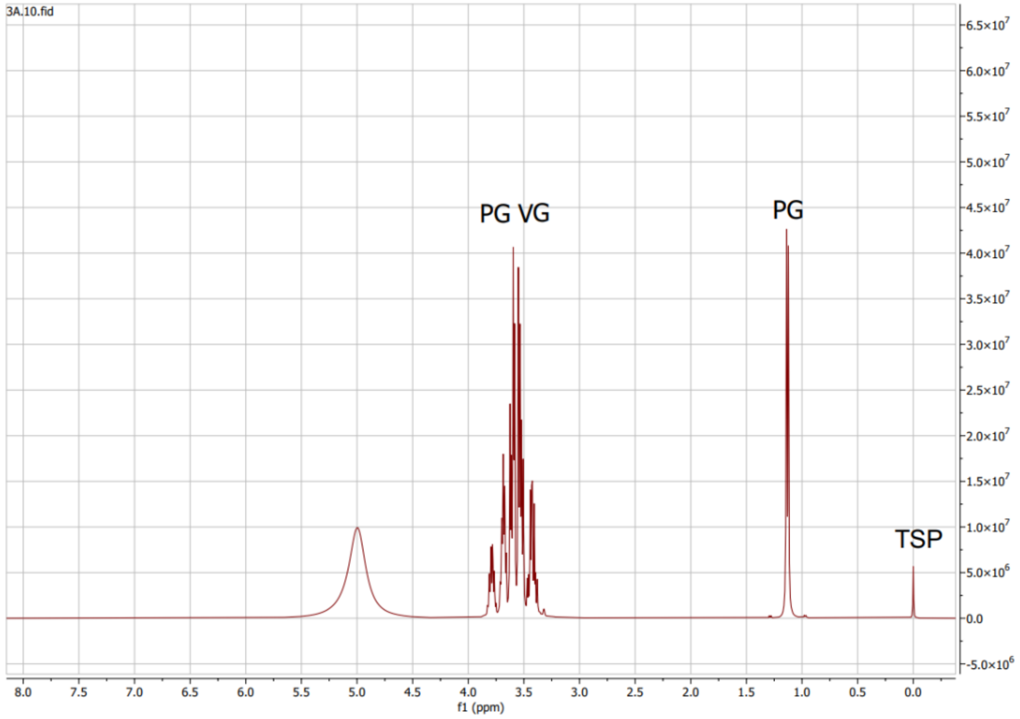

Figure S5. 1H NMR spectrum of exemplar police-seized SC-containing e-liquid sample. The reference standard trimethylsilyl propanoic acid (TSP), propylene glycol (PG) and glycerol (VG) peaks are labeled. Aromatic peaks arising from the indazole core of MDMB-4en-PINACA are not visible due to scaling.

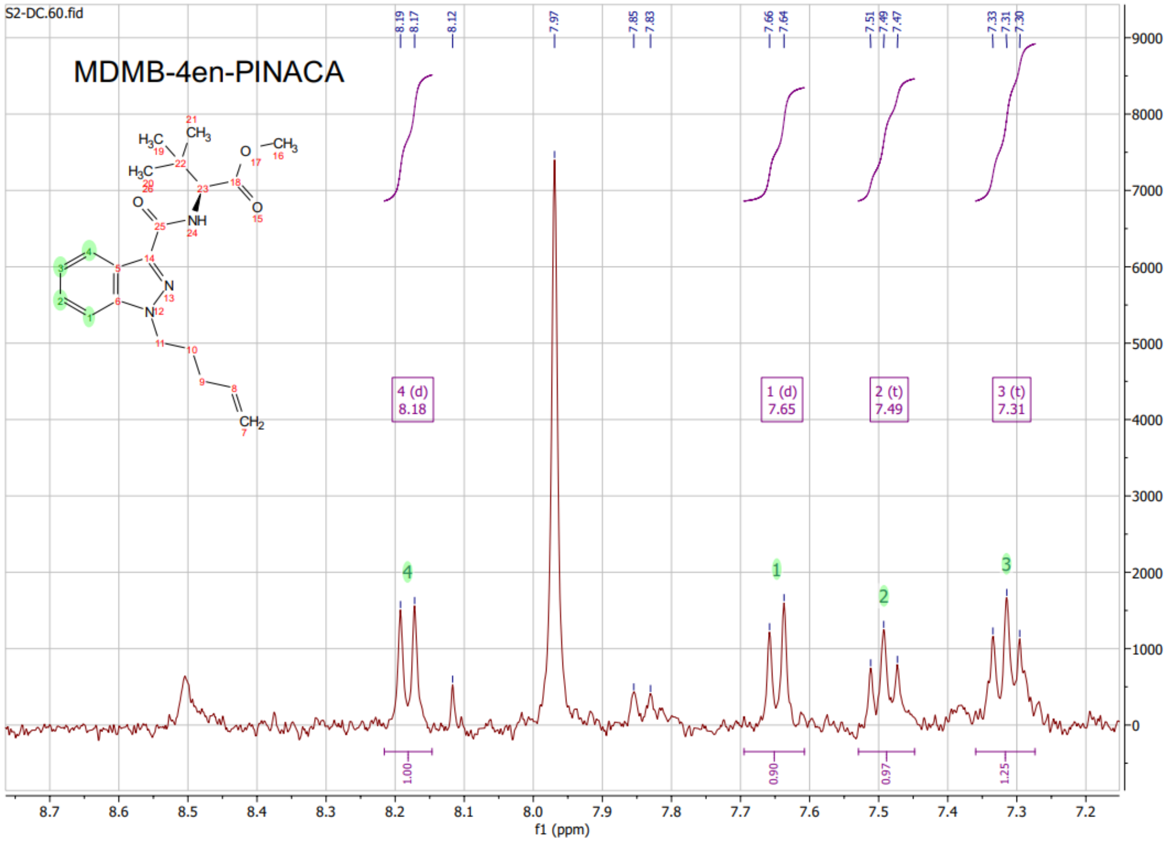

Figure S6. 1H NMR spectrum of police seized SC-containing e-liquid (Seizure 1). Four aromatic peaks used in quantification of MDMB-4en-PINACA are assigned in green and correspond to atom numbering in the accompanying molecular structure.

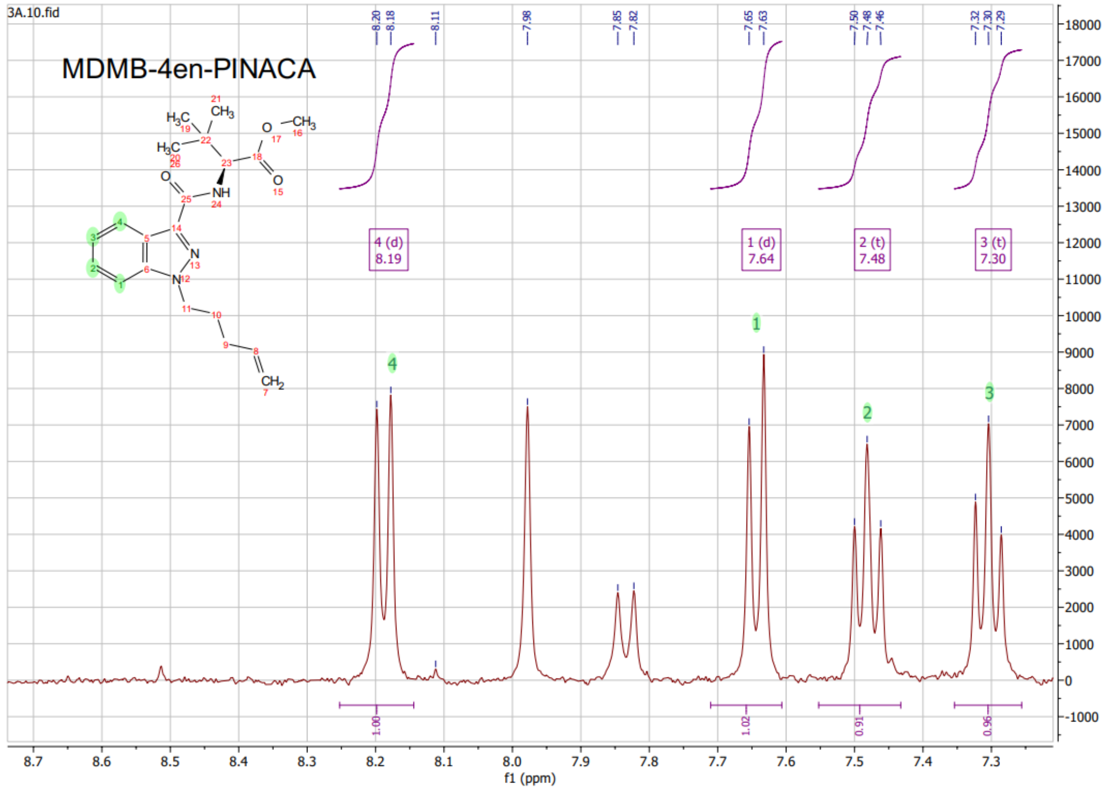

Figure S7. 1H NMR spectrum of police seized SC-containing e-liquid (Sample A, seizure 2). Four aromatic peaks used in quantification of MDMB-4en-PINACA are assigned in green and correspond to atom numbering in the accompanying molecular structure.

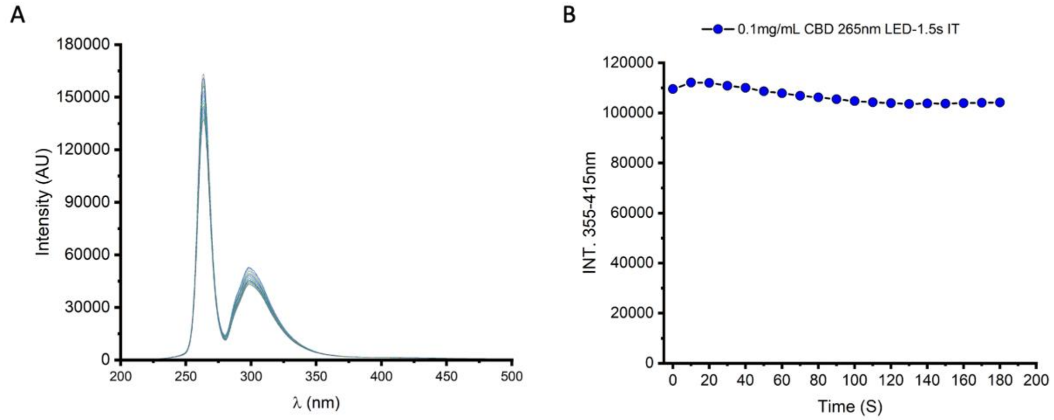

Figure S8. CBD is not photochemically reactive. (A), 1 mL 0.1 mg/mL CBD irradiated with 265 nm LED. 10 s increments, 1500 ms integration time. (B), Time-course integrated region of 355-415 nm showing no increase in signal magnitude as found with identical treatment of THC.

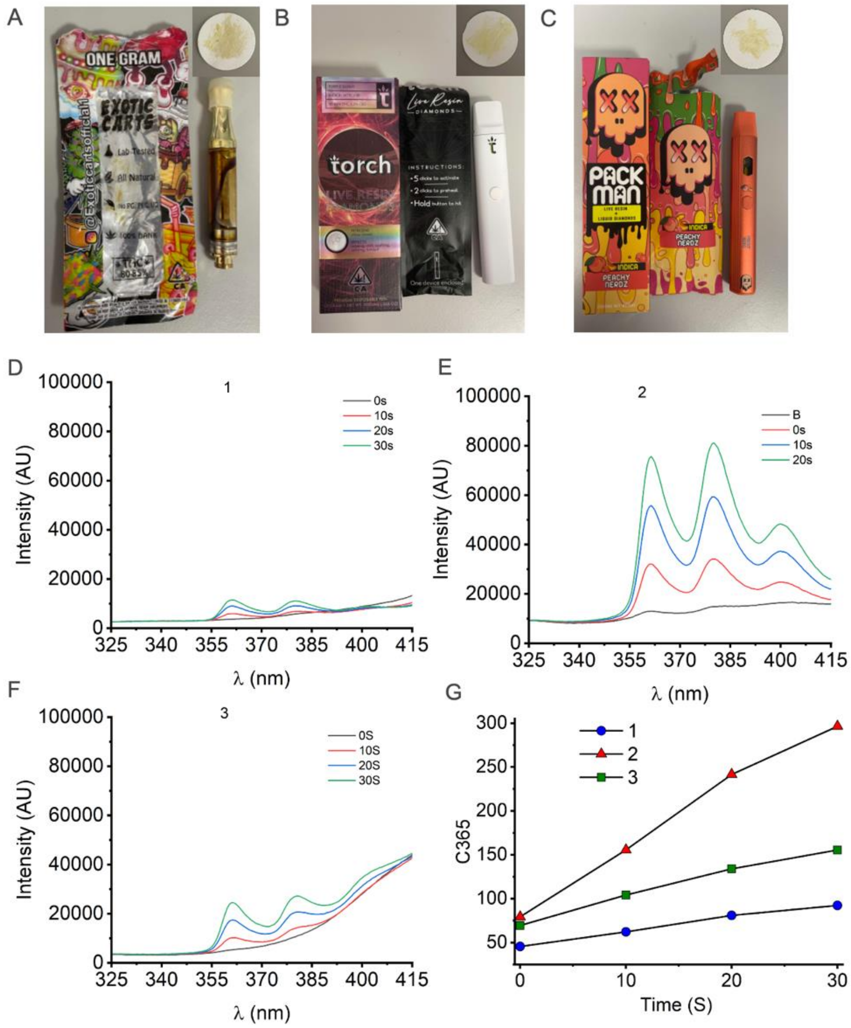

Figure S9. Validation of photochemical-based detection of THC. (A-C), Real-world cannabinoid resin e-cigarette cartridges containing, 80% (A), 90% (B), and unspecified (C) THC content. Whatman filter discs were prepared with 5mg resin extracts. (D-F), Direct spectral measurement of filters containing resin extract from A-C. Time course measurements taken in 10 s increments from t = 0-30 s. Continuous irradiation with 265 nm LED, integration time 4 s per measurement. (G), Time-course response of C365 obtained from device measurement of fresh material from samples A-C.

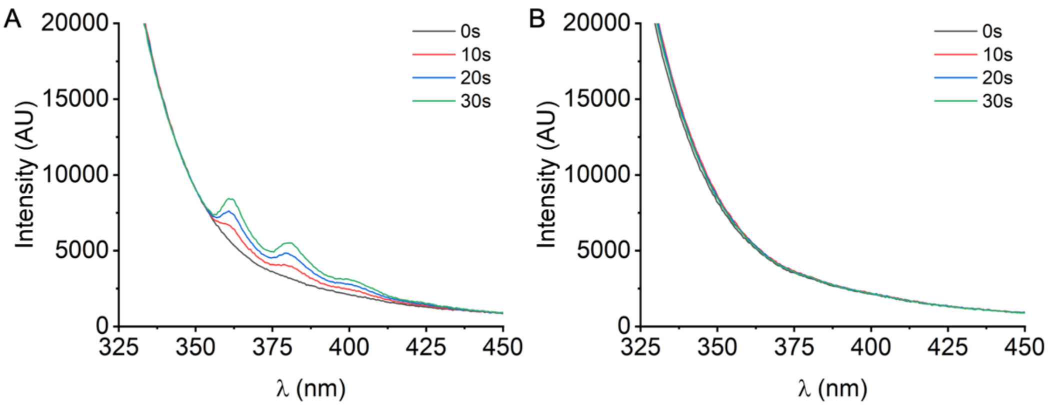

Figure S10. Photochemical reactivity of delta-8-THC and hexahydrocannabinol (HHC). (A), Delta-8-THC is photochemically reactive. 1mL 0.1 mg/mL delta-8-THC irradiated with 265 nm LED. 10 s increments, 5000 ms integration time. (B), Hexahydrocanabinol (HHC) is not photochemically reactive. 1mL 0.1 mg/mL HHC irradiated with 265 nm LED. 10 s increments, 5000 ms integration time.

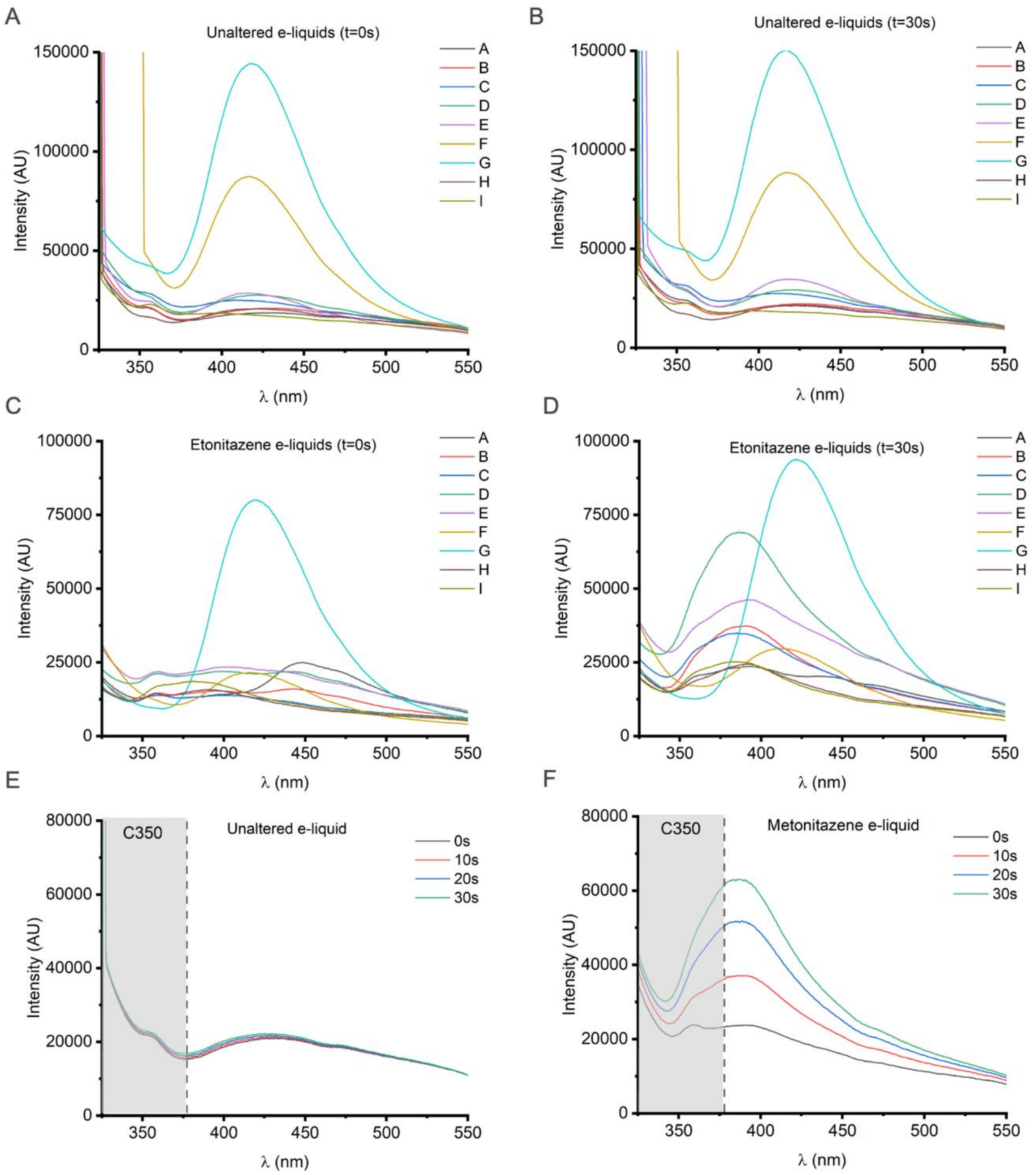

Figure S11. (A), Spectra of 9 commercially available e-liquids added to elf bars, artificially actuated by the device, and deposited onto filters. Measurements taken at t = 0 s. Irradiation with 265 nm LED, integration time 4 s. E-liquids corresponding to A-I are described in Table S3. (B), As in A, measurement taken after 30 s irradiation. (C), As in A, with etonitazene (freebase) present at 1.5 mg/mL. (D), As in C, measurement taken after 30 s irradiation. (E), Unaltered e-liquid (Liberty Flights, Purple Crush) added to elf bar, artificially actuated, and deposited onto filter. Time-course spectra taken at 10s intervals from t = 0-30 s. Integration time 4s per measurement. (F), As in E, with metonitazene (HCl) present at 1.5 mg/mL.

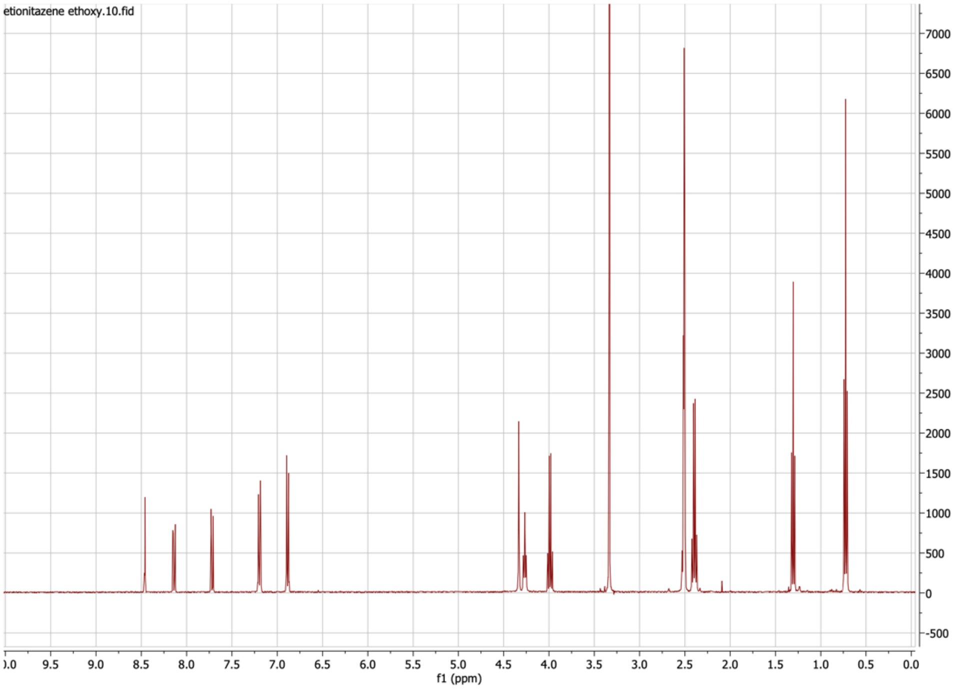

Figure S12.1H NMR spectrum of etonitazene, >95% pure.

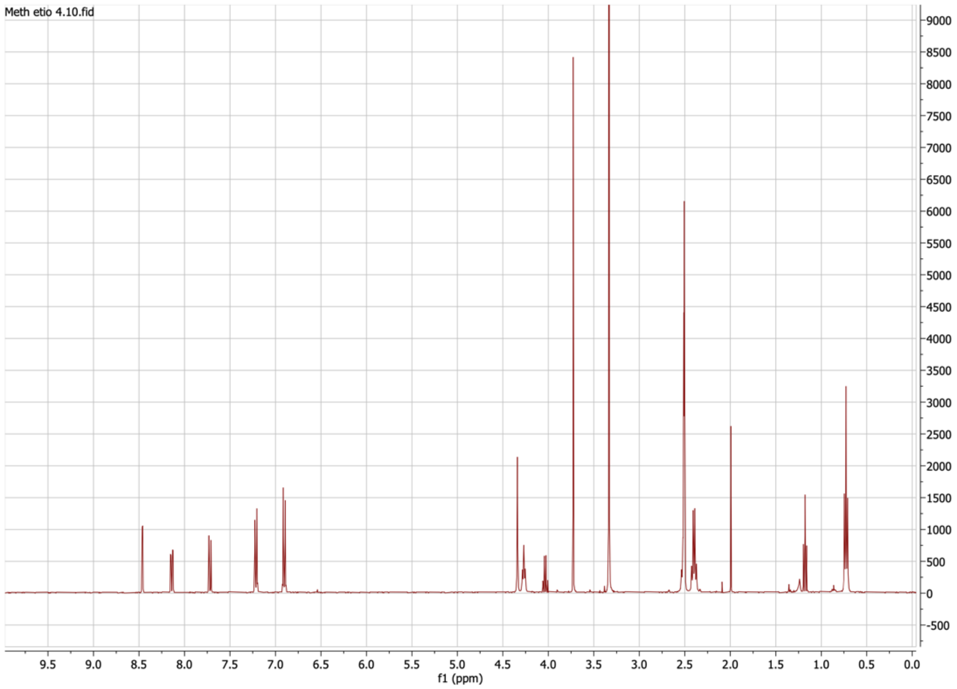

Figure S13.1H NMR spectrum of metonitazene, >95% pure.

Table S1. Brand, flavor, and nicotine content of e-liquids spiked with 1.5mg/mL MDMB-4en-PINACA.

Figure 3, B-C	Brand	Flavor	Nicotine content
A	R&M tornado 10000	Blue Razz	2%
B	R&M tornado 10000	Blueberry Cherry	2%
C	Magic bar MAX (4000)	Icy Pineapple	2%
D	McKesse (MK bar 7000)	Apple Peach & Pear	Nicotine free
E	Magic bar MAX (4000)	Mr. BLUE	2%
F	R&M tornado 10000	Gummy Drop	2%
G	Magic bar MAX (4000)	Grape Ice	2%
H	R&M tornado 10000	Mixed berries	2%
I	Elux Legend 3500	Pink Lemonade	2%
J	Superior Vapour	Forest Fruits	Nicotine free
K	Superior Vapour	Banana	Nicotine free
L	Superior Vapour	Raspberry	Nicotine free
M	Superior Vapour	Cherry	Nicotine free

Table S2. Brand, flavor, and nicotine content of e-liquids spiked with 1.5mg/mL MDMB-CHMICA.

Figure 3, D:	Brand	Flavor	Nicotine content
A	R&M tornado 10000	Blue Razz	2%
B	R&M tornado 10000	Blueberry Cherry	2%
C	R&M tornado 10000	Gummy Drop	2%
D	Superior Vapour	Virginia Tobacco	0.6%
E	Superior Vapour	Strawberry & Lime	0.6%
F	Cirro	Regular Tobacco	0.6%

Table S3. Brand, flavor, and nicotine content of e-liquids spiked with 1.5mg/mL Etonitazene.

Figure S10, A-D:	Brand	Flavor	Nicotine content
A	Liberty Flights	British Tobacco	1.2%
B	Liberty Flights	Cherry Menthol	0.6%
C	Superior Vapour	Virginia Tobacco	0.6%
D	Superior Vapour	Strawberry & Lime	0.6%
E	Liberty Flights	Strawberry	0.6%
F	Liberty Flights	Purple Crush	0.6%
G	Liberty Flights	Grape	0.6%
H	Cirro	Cherry Bomb	1.2%
I	Cirro	Regular Tobacco	0.6%
